# The complete chloroplast genome sequence of an invasive plant *Solanum rostratum* (Solanaceae)

**DOI:** 10.1080/23802359.2020.1714506

**Published:** 2020-01-14

**Authors:** Xiaojun Shi, Juan Qiu

**Affiliations:** Xinjiang Key Laboratory of Grassland Resources and Ecology and Ministry of Education Key Laboratory for Western Arid Region Grassland Resources and Ecology, College of Grassland and Environment Sciences, Xinjiang Agricultural University, Urümqi, People’s Republic of China

**Keywords:** *Solanum rostratum*, chloroplast genome, Illumina sequencing, phylogeny

## Abstract

*Solanum rostratum* is native to Neotropics and southwestern United States and considered as an invasive plant in Americas, Europe, Asia, Africa, and Australia. However, information on the chloroplast (cp) genome of this species is limited. In this study, we present the complete chloroplast genome sequence of *S. rostratum* obtained by high-throughput nextgeneration sequencing technology. The whole cp genome was 155,559 bp long and comprised 130 genes, including 85 protein-coding genes, 37 tRNA genes, and eight rRNA genes. The *S. rostratum* cp genome had a GC content of 37.76%. Based on the concatenated coding sequences of cp PCGs, phylogenetic analysis showed that Phylogenetic tree revealed that *S. rostratum* and *S. elaeagnifolium* are closely related to each other within the genus *Solanum.*

*Solanum rostratum* (Solanaceae) is an annual herb native to Mexico and considered as an invasive plant in Americas, Europe, Asia, Africa, and Australia with a high reproductive ability, strong seed dispersal ability, and efficient seed germination ability (Lin and Tan 2007; Wei et al. 2009, 2010; Eminniyaz et al. 2013). In China, this species is first found in Liaoning Province in northeast China in 1980s. Now, the species has spread across almost all regions of Northern China including Liaoning, Jilin, Beijing, Hebei, Shanxi, Xinjiang, and Inner Mongolia (Wei et al. 2007; He et al. 2011). It has been listed in the Checklist of the Invasive Plants in China (Wei and Yang 2013). This invasive species is treated as a noxious weed and causes livestock discouraged or poisoned as poisonous thorns cover the body of *S. rostratum* (Zhu et al. 2011). In this study, we characterized the complete chloroplast genome sequence of *S. rostratum* a resource for future genetic studies on this and other related species, which may provide valuable guidance for the utilization and management of *S. rostratum*.

Fresh leaves of *S. rostratum* were obtained from the Urumqi County, Urumqi, Xinjiang Province of China (87°46′E, 43°49′N) with voucher specimen deposited at the Xinjiang Agricultural University Herbarium (XN2019092603). After DNA extraction, high-throughput DNA sequencing (pair-end 150 bp) was conducted on an Illumina NovaSeq platform (Illumina, CA, USA) at Genepioneer Biotechnologies Inc., Nanjing, China. Approximately 5.0 Gb of sequence data were generated and used for the assembly of cp genome with SPAdes (Bankevich et al. 2012). The assembled genome was annotated using CpGAVAS (Liu et al. 2012). The cp genome of *Solanum demissum* (NC_041552.1) was included as the initial reference. The annotated genomic sequence has been submitted to GenBank with the accession number MN635796.

The complete chloroplast genome of *S. rostratum* which contained a typical conserved quadripartite structure, with a LSC region of 86,281 bp, a SSC region of 18,442 bp, and a pair of IRs regions of 25,418 bp, was 155,559 bp in length. The total GC content of whole genome, LSC, SSC, and IRa/IRb regions was 35.87, 31.91, 43.07, and 43.07%, respectively. A total of 130 genes were identified, including 85 protein-coding, 37 transfer RNA, and eight ribosome RNA genes. Among these genes, 19 genes (trnK-UUU, rps16, trnG-UCC, atpF, rpoC1, trnL-UAA, trnV-UAC, rps12, petB, petD, rpl16, rpl2 × 2, ndhB × 2, rps12, trnI-GAU × 2, trnA-UGC × 2, and ndhA) contained a single intron and two genes (ycf3 and clpP) contained two introns.

To identify the phylogenetic position of *S. rostratum*, phylogenetic analysis was conducted. The aligned complete chloroplast genome sequences of *S. rostratum* and 45 other species using MAFFT (Katoh and Standley 2013) were used for phylogenetic analysis. The neighbour-joining tree was constructed using MEGA 7.0 with 1000 bootstrap replicates (Kumar et al. 2016). Phylogenetic analysis showed that *S. rostratum* and *S. elaeagnifolium* are closely related to each other within the genus *Solanum* (Figure 1). The chloroplast resource may provide valuable guidelines for the management and utilization of *S. rostratum*.

**Figure 1.**
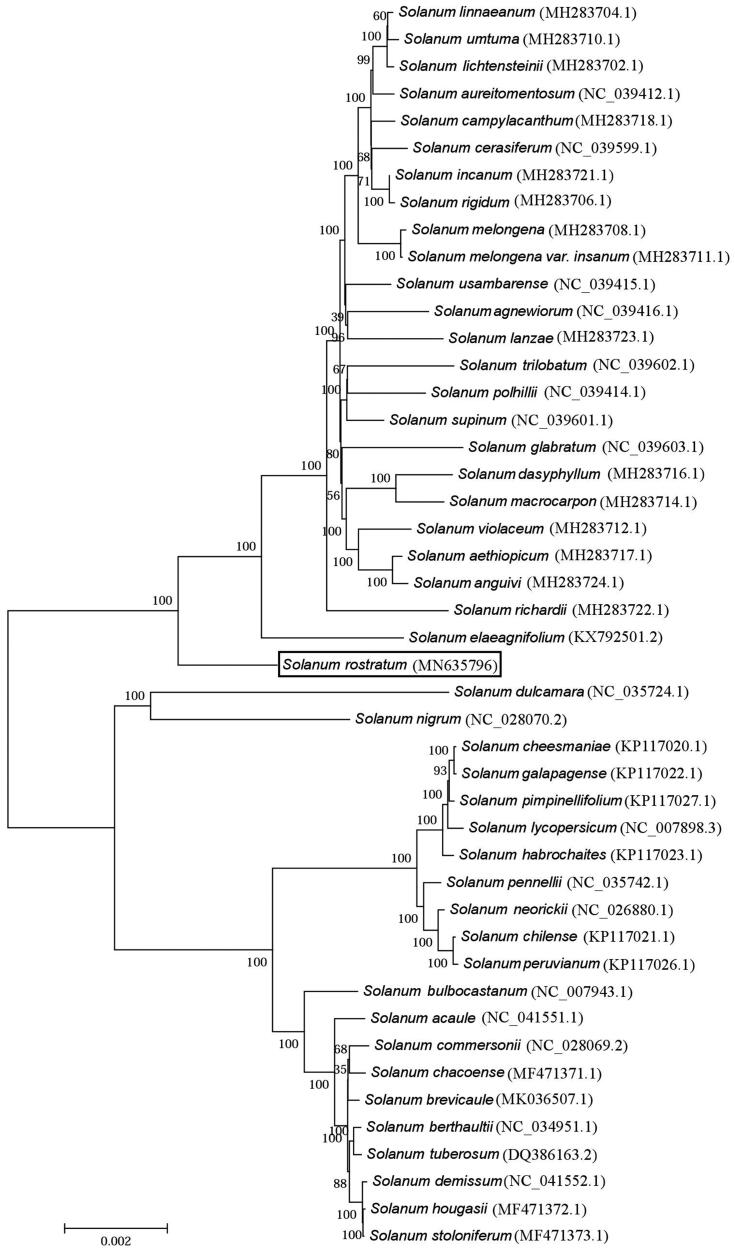
Phylogenetic tree based on the complete chloroplast genome sequences of *S. rostratum* and 45 other species belonging to the *Solanum*. The tree was generated using a neighbour-joining method using MEGA 7.0 with 1000 bootstrap replicates. Numbers on the nodes indicate bootstrap values. The NCBI accession numbers of chloroplast DNA sequences used in this study are presented in parentheses. The scale bar represents the number of substitutions per site.
